# Does In-Person Visiting Affect the Number of COVID-19 Cases in Prisons?

**DOI:** 10.3390/life11111184

**Published:** 2021-11-05

**Authors:** Lysandro Pinto Borges, Aline Fagundes Martins, Daniela Raguer Valadão de Souza, José Melquiades de Rezende Neto, Aryanne Araujo Santos, Brenda Morais Oliveira, Igor Leonardo Santos Matos, Grazielly Bispo da Invenção, Kezia Alves dos Santos, Nicolas Alessandro Alves Souza, Pamela Chaves de Jesus, Cliomar Alves dos Santos, Marco Aurélio de Oliveira Goes, Mércia Simone Feitosa de Souza, Ikaro Daniel de Carvalho Barreto, Adriana Gibara Guimarães, Lucindo José Quintans-Júnior

**Affiliations:** 1 Department of Pharmacy, Federal University of Sergipe, São Cristóvão 49100-000, Brazil; lysandro.borges@gmail.com (L.P.B.); ary_anne10@hotmail.com (A.A.S.); brenda@gmail.com (B.M.O.); igorleo@gmail.com (I.L.S.M.); grazybi@gmail.com (G.B.d.I.); keziaas@gmail.com (K.A.d.S.); nickl@gmail.com (N.A.A.S.); pamcjesus@gmail.com (P.C.d.J.); adrianagibara@hotmail.com (A.G.G.); 2 Department of Education in Health, Federal University of Sergipe, Lagarto 49400-000, Brazil; alinefm@gmail.com (A.F.M.); daniraguer@outlook.com (D.R.V.d.S.); jmr@gmail.com (J.M.d.R.N.); 3 Sergipe Central Public Health Laboratory, Aracaju 49015-460, Brazil; cliomaras@gmail.com; 4 State Health Department, Federal University of Sergipe, Aracaju 49060-108, Brazil; magoes@gmail.com (M.A.d.O.G.); merciafeitosa@gmail.com (M.S.F.d.S.); 5 Department of Statistics, Federal University of Pernambuco, Recife 50670-901, Brazil; ikarodcbarreto@gmail.com

**Keywords:** SARS-CoV-2, COVID-19, prison, penitentiary, visit

## Abstract

Even with the current advances that have been made in regard to COVID-19, such as a better understanding of the disease and the steady growth in the number of vaccinated individuals, it remains a challenge for humanity. Dealing with the disease in prison settings has been particularly difficult. This study sought to discover whether in-person visiting affected the number of cases of SARS-CoV-2 infection in the penitentiaries in the state of Sergipe (Brazil). We conducted a two-phase study (when visiting was suspended and after it recommenced) in seven penitentiaries in Sergipe using immunochromatography and nasopharyngeal swab testing to evaluate whether visiting affects the number of COVID-19 cases. In the first phase (*n* = 778), 57.6% of inmates reported risk factors and 32.5% were positive for COVID-19 (18.9% IgM, 24.2% IgG, 1% antigen). In the second phase, 19.6% tested positive (13.9% IgM, 7.9% IgG, 0.2% antigen). The occurrence of positive cases of COVID-19 and positive results (IgM and IgG) were significantly higher in the first phase. In the second phase, 56.7% of inmates had received visits and 18.7% were positive for COVID-19 (14% IgM, 7% IgG). Among those who had not received visits, 20.9% tested positive (13.8% IgM, 9.2% IgG, 0.5% antigen). There was no significant difference in positive cases/results between inmates that had and had not received visits. These findings suggest that, under the conditions assessed, visiting does not seem to affect the number of COVID-19 cases in prisons and reinforces the importance of sanitary measures to control dissemination.

## 1. Introduction

Since the confirmation of the first case of COVID-19 in Brazil on 26 February 2020 [1], there has been increasing concern about the spread of COVID-19 in the prison system. The Brazilian prison population is estimated to be over 760,000, and is the third largest in the world, behind only the US and China, in first and second place, respectively. Overcrowding (over 150% of capacity), associated with unsanitary conditions and the profile of inmates (characterized by the high prevalence of risk factors for COVID-19, such as cardiovascular, respiratory, metabolic, and immunosuppressive diseases; drug use; and unhealthy lifestyle habits, as well as the presence vulnerable groups such as older adults and pregnant women, among others), are conducive to the spread of the virus [2].

In Sergipe, a northeastern state of Brazil, the first case of COVID-19 was documented on 14 March 2020 [3]; however, our research group presented evidence that SARS-CoV-2 had circulated in the region before the first COVID-19 case was officially reported [4]. With regard to the state criminal institutions in Sergipe, the first case of COVID-19 was confirmed on 18 April 2020, and the first death on 6 May 2020 [5].

In order to reduce the risk of COVID-19 entering the prison system in Brazil, and considering the responsibility of the Brazilian justice system with regard to the protection of life and the guarantee of the dignity of this population, the National Council of Justice (CNJ), a public institution that aims to improve the work of the Brazilian judicial system, issued recommendation 62 on 17 March 2020. Its purpose was to guide the adoption of measures to prevent the spread of infection by the new coronavirus within the criminal and socio-educational justice systems [6]. The measures adopted by the Brazilian criminal justice system were in line with global guidelines and based on solid scientific evidence. These measures were grouped into five interdependent categories as follows: (1) recommendations focusing on risk groups, (2) reduction of population gatherings, (3) maintaining social distancing and/or social isolation, (4) management measures, and (5) acting on suspected cases [7]. However, some recommendations, such as social distancing and hand hygiene, have proven extremely difficult to enforce in the country’s prisons [8].

In Sergipe, as part of a contingency plan to control the spread of SARS-CoV-2 in the state penitentiary facilities, in-person visiting was banned on 18 March 2020 [9] and remained canceled until 14 September 2020 [10]. However, in prison, the suspension of family visits, combined with the perceived risk to health and life from COVID-19; the restrictions on circulation inside the prison walls; and the interruption of work, educational, and religious activities have significant emotional implications and tend to aggravate tensions [8,11].

In face of the apparent containment of COVID-19 among prisoners [10] during the period of suspension of visits and due to the importance of in-person visiting in regard to inmates’ mental health, the strengthening of family units and community ties, and the maintenance of public safety, this study sought to analyze whether in-person visiting affected the number of cases of SARS-CoV-2 infection in the penitentiary facilities in the state of Sergipe.

## 2. Methods

The study was approved by the National Bioethics Committee of Brazil (CAAE 31018520.0.0000.5546). Screening for SARS-CoV-2 infection involved inmates from seven prisons in the state of Sergipe, Brazil, and was performed in two phases (before and after in-person visiting recommenced). In the first phase (when in-person visiting was suspended), 1 or 2 inmates were randomly selected from each cell according to the number of residents. For each penitentiary facility, individuals from all cells were included in the study. Inmates presenting symptoms compatible with an acute respiratory infection were also included. Anti-SARS-CoV-2 IgM and IgG antibodies were identified using a finger-prick blood test-based or lateral flow sandwich detection immunochromatography (EGENS COVID-19 IgG/IgM Rapid Test Kit, Nantong Egens Biotechnology, Nantong China). Nasopharyngeal specimens were collected for SARS-CoV-2 antigen detection using an immunofluorescence assay (Eco F COVID-19 Ag kit with Eco Reader, Eco Diagnostica, Brazil) from inmates who presented symptoms or were asymptomatic and had a doubtful or positive result for IgM. All participants who tested positive for SARSCoV-2 antigens and/or IgM antibodies were submitted to cohort isolation for 14 days. First phase screening occurred from 31 August until 9 September 2020.

In the second phase (15 days after in-person visiting had recommenced), inmates who had tested negative for SARS-CoV-2 antigens and for IgM and IgG antibodies in the first phase were included in the study. Individuals who had tested positive for SARS-CoV-2 antigens and/or IgM and/or IgG antibodies in the first phase and those who were not at the prison facility at the time of testing were excluded from this phase. The same test procedures as those used in the first phase were performed. Second-phase screening occurred from 5 October until 9 October 2020.

All participants were enrolled in the study after giving their informed consent. Personal data including age, gender, presence of any risk factors, and symptoms compatible with COVID-19 were gathered by a health-care worker just before blood-sample collection.

Both rapid diagnostic tests applied in this study were performed at each penitentiary facility according to the manufacturer’s instructions. The EGENS COVID-19 IgG/IgM Rapid Test and the Eco F COVID-19 Ag showed, respectively, a sensitivity of 96.8% and 96.52%, and a specificity of 100% and over 99.9%, as reported by the manufacturer. Validation studies were performed for both methods using 26 and 24 samples, respectively. For the EGENS COVID-19 IgG/IgM Rapid Test Kit, we observed a sensitivity of 97% and a specificity of 99%. For the Eco F COVID-19 Ag, a sensitivity of 91.7% and a specificity over 99% were found.

### Statistical Analysis

The statistical data analysis was expressed as frequencies and percentages. Continuous variables were expressed as the mean and standard deviation when appropriate. A Z-test was performed in order to determine significant difference between proportions and a Z-Bonferroni test was conducted for pairwise proportions comparison. We also estimated unadjusted and adjusted odds ratios and their 95% confidence intervals with simple and multiple logistic regression. Statistical analyses were performed using Stata statistical software for data science (StataCorp., 2021, Stata Statistical Software: Release 17. StataCorp LLC, College Station, TX, USA).

## 3. Results

A total of 778 inmates were tested before in-person visiting recommenced and all participants provided consent. Of this sample, 692 (89%) were male and 86 (11%) were female. The mean age was 32 (SD 9.8) years old.

A total of 448 (57.6%) of the participants reported the presence of risk factors for COVID-19, such as smoking habits (30%); cardiovascular disease (12%), including systemic arterial hypertension, hypotension, cardiopathy, heart attack, and arrhythmia; respiratory disease (10%), including tuberculosis, asthma, pneumonia, and bronchitis, among others; diabetes mellitus (3%); high cholesterol (1%); HIV (0.5%); and anemia (0.5%).

**Figure 1:**
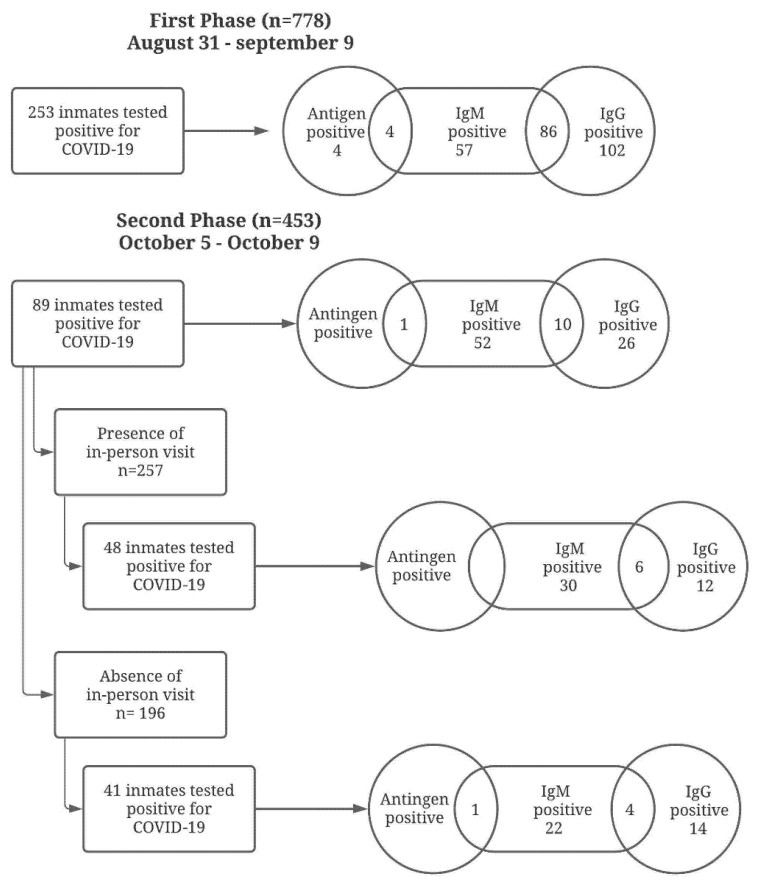
Flowchart of the study outcomes.

A flowchart outlining the study results is shown in Figure 1. Of the 778 inmates tested in stage one, 253 (32.5%) presented a positive result for COVID-19. A total of 147 (18.9%) had positive results for IgM (indicating an active/recent infection) and a total of 188 (24.2%) had positive results for IgG (indicating a past infection) (Table 1). Of these positive results, 86 were concomitantly positive for both IgM and IgG (IgM/IgG), indicating a recent infection that may still have been contagious. Only eight (1%) results were positive for SARS-CoV-2 antigens, and of these, four were also positive for IgM.

In the second phase (15 days after in-person visiting recommenced), inmates who tested positive for COVID-19 (253) in the first stage and those who were not at the prison facility at the moment of testing (71) were excluded from the study. A total of 453 inmates were tested after in-person visits recommenced and all participants provided consent. Of this sample, 396 (87.4%) were male and 57 (12.6%) were female. The mean age was 32.22 (SD 9.8) years old. Of the 453 inmates tested in the second phase, 89 (19.6%) showed a positive result for COVID-19 (Table 1). A total of 63 (13.9%) had positive results for IgM and a total of 36 (7.9%) had positive results for IgG (Table 1). Of these positive results, 10 were concomitantly positive for both IgM and IgG (IgM/IgG). Only one (0.2%) result showed positivity for SARS-CoV-2 antigens, and this result was also positive for IgM.

The occurrence of positive cases for COVID-19 was significantly higher in the first phase of the study (when in-person visiting was suspended) compared to the second phase (15 days after in-person visiting recommenced) (percent difference 12.9%, CI 7.8; 17.7%, *p* < 0.001). Similarly, the proportion of positive results for the antibodies against SARS-CoV-2 in the first phase of testing was significantly higher than those observed in the second phase (for IgM: percent difference 5%, CI 0.6; 9.1, *p* = 0.0277, and for IgG: percent difference 16.2%, CI 12.2; 20.0, *p* < 0.001) (Table 1).

In the second phase, 257 (56.7%) of the inmates tested had received in-person visits. Of the 257 who had received visits, 48 (18.7%) presented a positive result for COVID-19. One hundred ninety-six (43.3%) inmates had not received in-person visits. Of these 196, 41 (20.9%) tested positive for COVID-19. There was no significant difference in positive cases for COVID-19 between inmates that had and had not received in-person visits (percent difference −2.2%, CI −9.1; 5.1, *p* = 0.5530) in the second phase of testing (Table 2).

Of the group that had received in-person visits, a total of 36 (14%) positive results for IgM and a total of 18 (7%) positive results for IgG were found. Of these positive results, six were concomitantly positive for both IgM and IgG (IgM/IgG). There was no positive result for the SARS-CoV-2 antigen test. Of the group that had not received in-person visits, a total of 27 (13.8%) positive results for IgM and a total of 18 (9.2%) positive results for IgG were observed. Of these positive results, four were concomitantly positive for both IgM and IgG (IgM/IgG). Only one (0.5%) result showed positivity for SARS-CoV-2 antigens and this result was also positive for IgM. There was no significant difference in positive cases for IgM (percent difference 0.2%, CI −6.4; 6.5, *p* = 1.000) and IgG (percent difference −2.2%, CI −7.7; 2.8, *p* = 0.4836) antibodies between inmates that had received and had not received in-person visits in the second phase of testing (Table 2).

The seven penitentiary institutes studied were ranked according to their distribution of positive results for COVID-19 (Table 3). The three penitentiary institutes with the highest occurrence of SARS-CoV-2 infection in the state were the Territorial Jail of Nossa Senhora do Socorro, which was the penitentiary facility with the highest occurrence of positive cases for COVID-19, with 16 (30.8% of the total assessed inmates in that facility, 10 IgM-positive results (19.2%), 8 IgG-positive results (15.4%), and 2 IgM/IgG-positive results (3.8%)), followed by the Public Jail of Areia Branca, which presented 17 positive cases (25.76% of the total tested inmates in that facility, 13 IgM-positive results (19.7%), 7 IgG-positive results (10.6%), and 3 IgM/IgG-positive results (4.5%)) and the Public Jail of Estância, which presented 9 positive cases (20.5% of the total tested inmates in that facility, 4 IgM-positive results (9.1%), 6 IgG-positive results (13.6%), and 1 IgM/IgG-positive result (2.3%)). The female penitentiary facility (Female Prison) showed the lowest prevalence of infection, with 7 positive cases (9.9% of the total assessed inmates in that facility, 6 IgM-positive results (8.4%), 3 IgG-positive results (4.2%), and 2 IgM/IgG-positive results (2.8%)). A hypothesis to explain the different prevalence of infection observed among the penitentiary institutes may be the number of in-person visits each facility received (Table 4). Although it is not seen as causal evidence, the data shown in Table 3 and Table 4 do not provide any relation between the number of inmates that received in-person visits and the percentage of positive cases for COVID-19 when we applied the Z-Bonferroni test for pairwise proportions comparison except for the Presídio Regional Senador Leite Neto when compared with the Complexo Penitenciário Advogado Antônio Jacinto Filho (percent difference 41.1%, CI 24.5; 54.7, *p* < 0.001) and the Cadeia Pública de Areia Branca (percent difference 37.8%, CI 20.4; 51.4, *p* < 0.001).

In Table 5, we employed a multiple logistic regression relating positiveness and visits. We observed a significant association with an unadjusted odds ratio of 0.58 (CI 0.38–0.76, *p* = 0.001), indicating that visits lower the odds of positiveness. However, we adjusted this result with a number of confounders (age, sex, comorbidities) and observed no significant result with an adjusted odds ratio of 0.81 (CI 0.54–2.88, *p* = 0.311).

## 4. Discussion

In order to combat the spread of SARS-CoV-2 infection in prisons, the WHO provided global guidelines with a number of recommendations that included access restriction (the suspension of visits) and movement limitations [12]. In Sergipe, Brazil, the suspension of visits in the state penitentiary facilities started on 18 March 2020 [9] and remained in place until 14 September 2020 [10]. However, beyond the question of whether visits make COVID-19 outbreaks in prisons more likely, such restrictive measures may have a profound impact on the inmates’ mental health, inducing major stress among incarcerated populations and aggravating tensions [8,11,13]. Stopping visits could also affect the inmates’ general health and hygiene, favoring the spread of diseases, since, due to the inability of authorities to properly feed inmates, many inmates rely on food and medicine brought in by relatives [14].

Due to the importance of in-person visiting for inmates’ mental and general health, and in regard to public safety, we conducted a two-phase study in seven penitentiary facilities in the state of Sergipe to address the question of whether in-person visiting does actually affect the number of SARS-CoV-2 cases in prisons.

In the first phase of the study (when visiting was suspended), 778 inmates were tested for COVID-19. Among those tested, 57.6% reported the presence of risk factors for the disease, despite the low mean age (32 years old) observed. Smoking habits (30%), cardiovascular disease (12%), and respiratory disease (10%) were the three risk most commonly reported risk factors by inmates. A number of studies have shown that incarcerated populations have an increased prevalence of chronic medical conditions and infectious diseases, with percentages far greater than those for the population at large, even when comparing similar age groups [15,16,17]. These data are of particular importance since comorbidities have been directly associated with the severity of the COVID-19 disease [18]. Indeed, many inmates are smokers, drug users, and are obese with heart and respiratory illnesses, and, therefore, are already at risk of infection from a variety of diseases whose transmission tends to be boosted by the unsanitary conditions found in prisons [14].

The infrastructure of most prisons and jails is also conducive to the spread of disease. as social distancing is typically a physical impossibility [19]. In Brazil, as in other Latin American countries, prisoners are locked in damp, overcrowded, and poorly ventilated cells with limited access to drinking water. They also share bathrooms and living spaces, as well as personal hygiene items [14]. Hence, prisoners are probably more vulnerable to COVID-19 than the general population due to the confined environment in which they live, which can act as a source of infection and amplify the spread of infectious diseases [20].

Brazil, taking into account the experience of the pandemic in other countries, implemented various initiatives to reduce the spread of the virus in prisons. The National Penitentiary Department mandated the suspension of the entry of food and visitors; relaxed prison sentencing, especially for those at risk; and instituted the use of videoconferences for social, legal, educational, and religious contact [21]. Other measures adopted to mitigate COVID-19 transmission included the mandatory use of masks in prisons, the promotion of increased personal hygiene and environmental sanitizing, decontamination and disinfection of transfer vehicles, a 14-day isolation period for new prisoners, and limiting the transfer of prisoners between facilities [9].

However, despite these targeted efforts to contain viral dissemination, our first phase outcomes revealed that 32.5% of the inmates presented a positive result for COVID-19, showing that SARS-CoV-2 was circulating among prisoners. Our findings demonstrate that the total of positive results for IgM and IgG was 18.9% and 24.2%, respectively. Since in-person visiting had been suspended for almost six months when the first phase of this study was conducted, we assume that penitentiary staff were responsible for transmitting the virus, as correctional officers and other staff frequently leave the facility and then return [19].

Moreover, COVID-19 could be introduced to the penitentiary system through staff, visitors, or inmates under a semi-open regime. Infections can be transmitted between prisoners, staff, and visitors; between prisons through transfers and staff cross-deployment; and to and from the community [22]. In addition, the great number of infected persons reporting no symptoms [23,24,25,26] makes infection difficult to track. A study conducted by our research group in May 2020 showed a high prevalence of SARS-CoV-2 antibodies among asymptomatic individuals in the state of Sergipe, Brazil [27]. Based on these factors, it was reasonable to expect an increase in the number of COVID-19 cases among inmates after in-person visiting recommenced. However, our data revealed that of the 453 inmates tested in the second phase, 19.6% showed a positive result for COVID-19, with 13.9% and 7.9% presenting positive results for IgM and IgG, respectively. The statistical analysis shows that the occurrence of positive cases for COVID-19 was significantly higher in the first phase of the study (when in-person visits were suspended) compared to the second phase (15 days after in-person visiting recommenced). Likewise, the proportion of positive results for the antibodies against SARS-CoV-2 in the first phase was significantly higher than those found in the second phase, so we hypothesize that the significant differences observed were due to the improved sanitary measures that had been put in place after the first phase of testing, including cohort isolation of confirmed positive cases. In fact, in the context of overcrowded prisons and given the impossibility of mandating effective social distancing, implementing broad testing strategies to promptly identify COVID-19 and rapidly block transmission are considered fundamental for accurately measuring and preventing viral spread and planning better interventions [8,28,29]. Prison systems should be the focus of robust testing efforts and be provided with the resources to develop and deploy long-term testing strategies [30], since evidence from mass testing in prisons supports the idea of faster viral spread, with the majority of cases identified being pre-symptomatic or asymptomatic [28,31].

In the second phase of testing, we compared inmates that had and had not received in-person visits. Our findings showed no significant difference in positive cases for COVID-19 and for positive results for IgM and IgG antibodies between inmates who had and had not received in-person visits. These data suggest that in-person visiting does not affect the number of cases of SARS-CoV-2 infection in prisons. We also ranked the seven penitentiary institutes studied according to their distribution of positive results for COVID-19 and the number of in-person visits each facility received. The Territorial Jail of Nossa Senhora do Socorro, the Public Jail of Areia Branca, and the Public Jail of Estância were the three penitentiary institutes with the highest occurrence of SARS-CoV-2 infection in the state, whereas the female penitentiary facility showed the lowest number of positive cases. Regarding the distribution of visits, the Penitentiary Complex of Advogado Antônio Jacinto Filho, the Public Jail of Areia Branca, and the Territorial Jail of Nossa Senhora do Socorro were the three penitentiary facilities with the highest number of in-person visits. The Regional Prison Senador Leite Neto displayed the lowest number of visits. Our results do not provide any evidence of a relationship between the number of inmates that received in-person visits and the percentage of positive cases for COVID-19. Together, these findings corroborate the evidence that in-person visiting does not affect the number of positive cases for SARS-CoV-2 in prisons. The outcomes found in the second phase of the study may be explained by the preventive measures adopted as part of a contingency plan involving changes in the regime of visits, including in regard to the delivery of items to inmates, the mandatory cleaning of visitation spaces, the provision of masks and individual protection items to visitors, prohibiting the entry of visitors with fever or respiratory symptoms associated with COVID-19, and spreading visiting times over more days and over longer periods of the day to reduce the concentration of visitors [5,6]. Our results reinforce the importance of sanitary measures to control the entrance and transmission of COVID-19 into the penitentiary system. In fact, the effectiveness of the protocol established by the WHO regarding the COVID-19 pandemic and the procedures for preventing its spread in the prison environment [12] was shown in a study conducted in the penitentiary facilities in the province of Salerno, Italy, where serological and nasopharyngeal swab screening executed on inmates and personnel working in prisons showed no positive results for COVID-19 [20].

This study has some limitations. First, occurrence estimates may change with new information on the accuracy of the test kits used. Second, we cannot completely rule out the possibility that there is a link between the number of positive cases for COVID-19 and the number of in-person visits. One of the characteristics of the prison system in Sergipe is that the cells have a capacity for six prisoners, and when the visiting period is open, they can receive one visitor per month, with only people without symptoms being allowed to enter the prison system wearing an N95 mask, and during the visit, there is no contact between the prisoner and the visitor.

The current study attempted to evaluate whether in-person visiting affected the number of cases of SARS-CoV-2 infection in the penitentiary facilities in the state of Sergipe, Brazil. Our data suggest that in-person visiting does not affect the number of positive cases of COVID-19 and reinforce the importance of mass testing and following preventive and control measures in order to safeguard prison-system health and public health, especially as prisons may act as reservoirs that could lead to a resurgence of the pandemic [17,32]. Moreover, the population in vulnerable situations are historically more susceptible to the serious repercussions imposed by COVID-19 outbreak, especially the black population [33], which makes up about 80% of the entire prison population in Sergipe. Studies like this one are necessary to assist global policymakers to plan and implement measures that could lead to a successful experience in dealing with the pandemic in prison systems.

## Figures and Tables

**Table 1:** Occurrence of SARS-CoV-2 infection before and after in-person visit recommenced in the penitentiary facilities in the state of Sergipe, Brazil, August–October 2020.

Phase	Total of Inmates with a Positive Result	Total of IgM-Positive Results	Total of IgG-Positive Results	Total of IgM/IgG-Positive Results	Total of Antigen-Positive Results
Before in-person visiting	253 (32.5%)	147 (18.9%)	188 (24.2%)	86 (11%)	8 (1%)
After in-person visiting	89 (19.6%)	63 (13.9%)	36 (7.9%)	10 (2.2%)	1 (0.2%)
% difference	12.9	5.0	16.2	8.8	0.8
95% CI	7.8; 17.7	0.6; 9.1	12.2; 20.0	6.1; 11.4	−0.3; 1.8
*p*-value	<0.001	0.0277	<0.001	<0.001	0.1667

Note: Z-test for proportions.

**Table 2:** Occurrence of SARS-CoV-2 infection in individuals who had and had not received in-person visits in the penitentiary facilities in the state of Sergipe, Brazil, October 2020.

Variable	Total of Inmates with a Positive Result	Total of IgM-Positive Results	Total of IgG-Positive Results	Total of IgM/IgG-Positive Results	Total of Antigen-Positive Results
Presence of in-person visiting	48 (18.7%)	36 (14%)	18 (7%)	6 (2.3%)	0
Absence of in-person visiting	41 (20.9%)	27 (13.8%)	18 (9.2%)	4 (2%)	1 (0.5%)
% difference	−2.2	0.2	−2.2	0.3	−0.5
95% CI	−9.1; 5.1	−6.4; 6.5	−7.7; 2.8	−3.0; 3.2	−2.8; 1.0
*p*-value	0.5534	1.0000	0.4836	1.000	0.4327

Note: Z-test for proportions.

**Table 3:** Rank of distribution of positive cases for SARS-CoV-2 infection in the seven penitentiary facilities in the state of Sergipe, Brazil, October 2020.

Penitentiary Facility	Total of Inmates Tested	Total of Inmates with a Positive Result	Total of IgM-Positive Results	Total of IgG-Positive Results	Total of IgM/IgG-Positive Results	Total of Antigen-Positive Results
Territorial Jail of Nossa Senhora do Socorro	52	16 (30.8%)	10 (19.2%)	8 (15.4%)	2 (3.8%)	0
Public Jail of Areia Branca	66	17 (25.8%)	13 (19.7%)	7 (10.6%)	3 (4.5%)	0
Public Jail of Estância	44	9 (20.5%)	4 (9.1%)	6 (13.6%)	1 (2.3%)	0
Regional Prison Senador Leite Neto	65	13 (20%)	9 (13.8%)	6 (9.2%)	2 (3.0%)	0
Penitentiary Complex Advogado Antônio Jacinto Filho	68	12 (17.6%)	11 (16.2%)	1 (1.5%)	0	0
Regional Prison Juiz Manoel Barbosa de Souza	87	15 (17.2%)	10 (11.5%)	5 (5.7%)	0	0
Female Prison	71	7 (9.9%)	6 (8.4%)	3 (4.2%)	2 (2.8%)	1 (1.4%)

Note: Z-Bonferroni test for pairwise proportions comparison.

**Table 4:** Rank of distribution of inmates who received in-person visits in the seven penitentiary facilities in the state of Sergipe, Brazil, October 2020.

Penitentiary Facility	Total of Inmates Tested	Total of Inmates Who Received in-Person Visits
Complexo Penitenciário Advogado Antônio Jacinto Filho	68	51 (75%)
Cadeia Pública de Areia Branca	66	47 (71%)
Cadeia Territorial de Nossa Senhora do Socorro	52	30 (57.7%)
Presídio regional Juiz Manoel Barbosa de Souza	87	49 (56%)
Presídio Feminino	71	38 (53.2%)
Cadeia Pública de Estância	44	20 (45.5%)
Presídio Regional Senador Leite Neto	65	22 (33.8%)

Note: Z-Bonferroni test for pairwise proportions comparison.

**Table 5:** Association between positive COVID-19 results and receiving visits among inmates.

	Inmates with a Positive Result					
	OR	95% CI	*p*-value	ORa	95% CI	*p*-value
Received visit	0.58	0.38–0.76	0.001	0.81	0.54–2.88	0.311

Note: In the adjusted model, we used as confounders age, sex, and comorbidity or the presence of single diseases. Ora: adjusted odds ratio.
